# Global trends in research related to social media in psychology: mapping and bibliometric analysis

**DOI:** 10.1186/s13033-018-0182-6

**Published:** 2018-01-19

**Authors:** Sa’ed H. Zyoud, Waleed M. Sweileh, Rahmat Awang, Samah W. Al-Jabi

**Affiliations:** 1 Poison Control and Drug Information Center (PCDIC), College of Medicine and Health Sciences, An-Najah National University, Nablus, 44839 Palestine; 2 Department of Clinical and Community Pharmacy, College of Medicine and Health Sciences, An-Najah National University, Nablus, 44839 Palestine; 3 Department of Pharmacology and Toxicology, College of Medicine and Health Sciences, An-Najah National University, Nablus, 44839 Palestine; 4 WHO Collaborating Centre for Drug Information, National Poison Centre, Universiti Sains Malaysia (USM), 11800 Penang, Malaysia

**Keywords:** Social media, Facebook, Twitter, Linkedin, Snapchat, Instagram, Bibliometric, Psychology

## Abstract

**Background:**

Social media, defined as interactive Web applications, have been on the rise globally, particularly among adults. The objective of this study was to investigate the trend of the literature related to the most used social network worldwide (i.e. Facebook, Twitter, LinkedIn, Snapchat, and Instagram) in the field of psychology. Specifically, this study will assess the growth in publications, citation analysis, international collaboration, author productivity, emerging topics and the mapping of frequent terms in publications pertaining to social media in the field of psychology.

**Methods:**

Publications related to social media in the field of psychology published between 2004 and 2014 were obtained from the Web of Science. The records extracted were analysed for bibliometric characteristics such as the growth in publications, citation analysis, international collaboration, emerging topics and the mapping of frequent terms in publications pertaining to social media in the field of psychology. VOSviewer v.1.6.5 was used to construct scientific maps.

**Results:**

Overall, 959 publications were retrieved during the period between 2004 and 2015. The number of research publications in social media in the field of psychology showed a steady upward growth. Publications from the USA accounted for 57.14% of the total publications and the highest *h*-index (48).The most common document type was research articles (873; 91.03%). Over 99.06% of the publications were published in English. *Computers in Human Behavior* was the most prolific journal. The *University of Wisconsin*–*Madison* ranked first in terms of the total publications (n = 39). A visualisation analysis showed that personality psychology, experimental psychology, psychological risk factors, and developmental psychology were continual concerns of the research.

**Conclusions:**

This is the first study reporting the global trends in the research related to social media in the psychology field. Based on the raw data from the Web of Science, publication characteristics such as quality and quantity were assessed using bibliometric techniques over 12 years. The USA and its institutions play a dominant role in this topic. The most preferred topics related to social media in psychology are personality psychology, experimental psychology, psychological risk factors, and developmental psychology.

## Background

Social media, defined as interactive Web applications [1], have been on the rise globally, particularly among adults [2, 3]. Overall, Facebook, Twitter, LinkedIn, Snapchat, and Instagram were the most used social network worldwide [4, 5]. Hundreds of publications have discussed the benefits and harm stemming from social media in different age groups of both genders [6]. Of particular interest is the impact of social media on the psychology and self-image of users. A recently published report indicated that social media can be used to forecast and prevent suicide attempts at the national level [7]. Another recent report indicated that social media, particularly Facebook, are positively correlated with divorce [8]. Survey studies, such as that conducted by Clayton et al. [9], had shown that high levels of Facebook use, when mediated by Facebook-related conflict with romantic partners, significantly predict negative relationship outcomes [9–11]. Previous studies clearly indicated that social media makes it easy for users to reconnect with any past lover, which could lead to emotional cheating and this could then lead to a breakup or divorce [9–11]. The diverse psychological and behavioral effects of social media on users necessitate further and deeper analysis. Such an analysis will be of value not only to academic researchers, but also to sociology experts, psychologists, psychiatrists, and even to those in the field of telecommunications to adapt and tailor these social media to the psychological health and needs of the users. Bibliometric and scientometric studies on Facebook and other social media have been carried out to assess the research trends in these social media in general [12–15]. Similarly, several bibliometric and scientometric studies have been accomplished to assess the research trends in psychology and behavior [16–18]. However, no search of the literature for bibliometric or scientometric analyses of psychology publications pertaining to social media was found. In response, this study was designed to address this gap by mapping the literature regarding the largest and most popular social media (i.e. Facebook, Twitter, LinkedIn, Snapchat, and Instagram) [4, 5] in the field of psychology. Specifically, this study will assess the growth in publications, citation analysis, international collaboration, author productivity, emerging topics and the mapping of frequent terms in publications pertaining to social media in the field of psychology.

## Methods

The database used in this bibliometric study, the Web of Science (WoS) database: Core Collection [20], is one of the largest and comprehensive bibliographic databases covering multidisciplinary areas. It encompasses over 12,000 of the highest impact journals worldwide (i.e. those considered to be highly influential in their fields) that contain somewhat higher data quality in the scientific, technical, medical, and social sciences [19–23].

To retrieve the research related to social media in the field of psychology, we applied the following steps to conduct this bibliometric study:

Step 1: The topic search query phrase “(Facebook OR Twitter OR LinkedIn OR Snapchat OR Instagram)” was applied to gather all the publications with those phrases in their titles, abstracts, or keywords. The documents published during the period from 2004 to 2015 were included in the study while the years 2016 and 2017 were excluded, as those years are still open for new issues.
Step 2: We then limited our retrieved publications related to social media to all those indexed under research categories related to psychology in the WoS database, including “Psychology”, “Psychology Clinical”, “Psychology Developmental”, “Psychology Multidisciplinary”, “Psychology Experimental”, “Psychology Social”, and “Psychology Applied”.
Step 3: The search queries from steps 1 and 2 were merged into one search query as the following: TS = (Facebook) OR TS = (Twitter) OR TS = (LinkedIn) OR TS = (Snapchat) OR TS = (Instagram). It was refined by the following Web of Science categories:(Psychology Clinical OR Psychology OR Psychology Developmental OR Psychology Multidisciplinary OR Psychology Experimental OR Psychology Social OR Psychology Applied).
Step 4: All the collected data were analysed and plotted based on the following characteristics: publication year, the main journal in the field, the institutions, the country/territory, the document type and language, the *h*-index, the impact factor (IF), the collaboration, and the citations. The IF was used according to the 2015 Journal Citation Reports^®^ published by Thomson Reuters, 2016 [24].
Step 5: We then analysed the records to identify the relationship between the countries, the institutions, and the terms through visualisations the main clusters in each one by using VOSviewer v.1.6.5 software. The key terms were recognised in the titles and abstracts of the retrieved publications related to social media in psychology, and the co-occurrence frequencies of these terms were calculated. The term map was constructed based on the co-occurrence frequencies of these terms to cluster the main topics in this field.

### Statistical analysis

Microsoft Excel 2007 and VOSviewer v.1.6.5 software were used for the graphics. SPSS statistical software (SPSS for Windows, version 16) was used for the statistical analyses. The Pearson correlation test was used to determine the correlation between the number of publication in the field of social media and the number of publication related to social media in psychology. P values < 0.05 were considered to be statistically significant. The descriptive statistics were presented as frequencies and percentages.

## Results and discussion

**Fig. 1:**
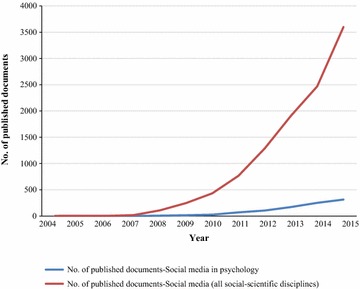
Yearly number of publications related to social media in psychology

The present study outlines the bibliometric indicators of the scientific research related to social media in psychology during the research timeframe from 2004 to 2015. We found a total of 10,843 publications related to social media published between 2004 and 2015. There were 959 scientific publications related to social media in psychology. Nine types of documents were found, and the most common was research articles (873 documents), which accounted for 91.03% of the total publications. The second most common document type was meeting abstracts (41 documents, 4.28% of the total). As expected, the majority of the publications were written in English (99.06%). Figure 1 shows the publication productivity related to social media and the publication productivity related to social media in psychology over time. The correlation analysis clearly shows a high correlation between the number of publication in the field of social media and the publication productivity related to social media in psychology (r = 0.995; p value < 0.001).

This increase in publications related to social media in psychology seems to be related to (1) social networking sites, which became very popular in previous decade [25, 26]; (2) increasing interest in this field in multiple health disciplines [27–31]; (3) or general cause such as the increasingly prevalent use of the internet, which has allowed more rapid distribution of medical knowledge through scientific research [32].

**Fig. 2:**
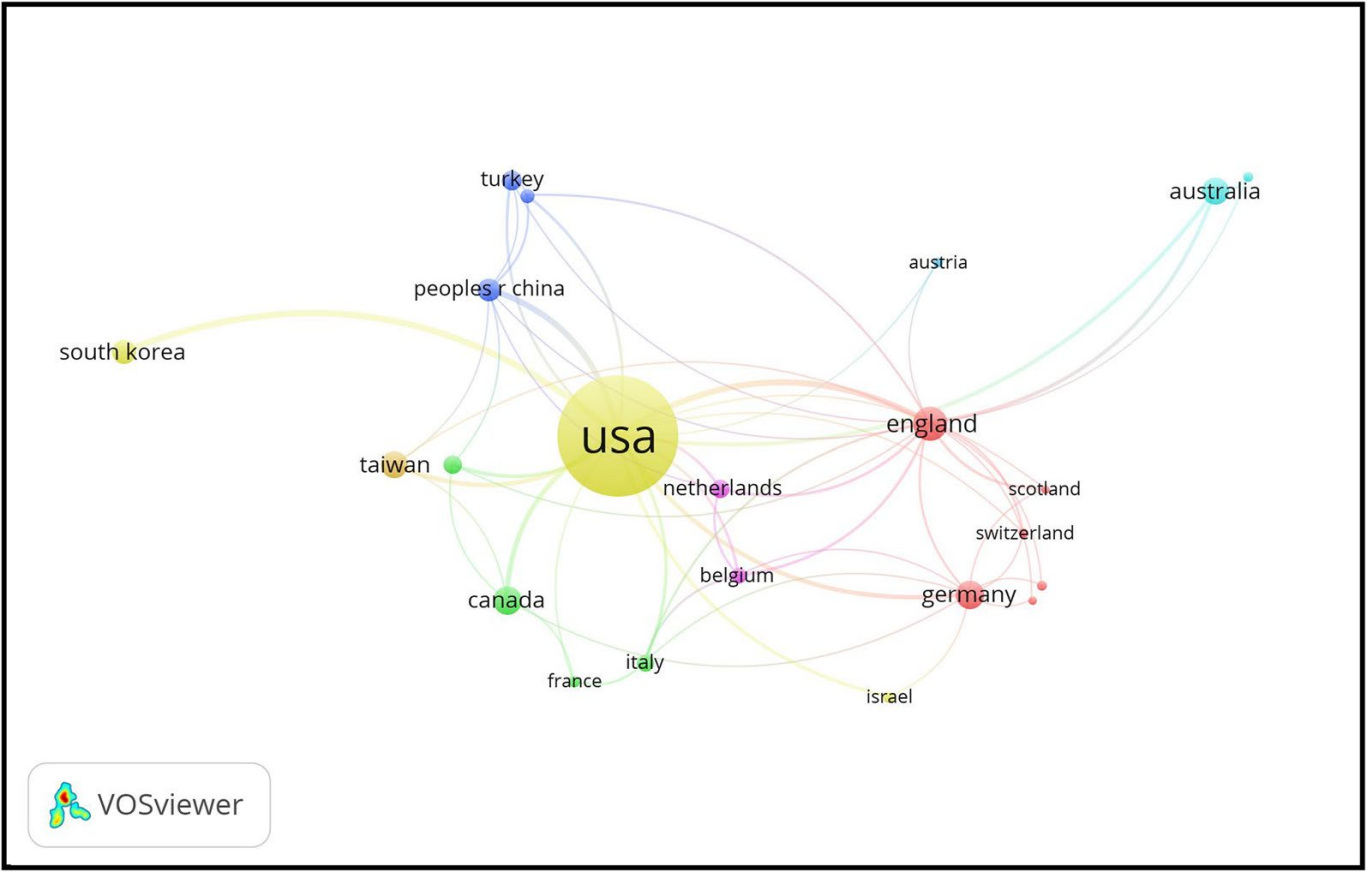
Combined mapping and clustering of productive countries/territories

Table 1 shows information about the 10 countries with the most published papers. The USA is responsible for the most papers (548 documents, 57.14% of the total), with the highest *h*-index values (*h*-index value = 48). The USA is followed by the UK (69, 6.69%), Canada (46, 4.80%), Germany (46, 4.80%), and Australia (44, 4.59%). The *h*-index for all retrieved publications was 59. The publications with the most international collaboration were those from the USA (n = 100). The UK and China ranked second and third, with 35 and 17 documents for each, respectively. The collaboration between countries based on co-authorship is shown in Fig. 2. The USA is the most networked country, collaborating with 26 countries, followed by the UK (n = 22) and Germany (n = 15). The USA was the most prolific country in producing publications related to psychology in social media, contributing to more than of half of the publications related to this topic. This research output from the USA is possibly due to the large size of the population or economic forces [33, 34]. Furthermore, most of the social networking sites were established and founded in the USA. These findings are similar to those found in previous bibliometric studies in different fields, principally that the USA had the highest activity in scientific research output worldwide and in international collaboration networks, as well as the highest *h*-index [32, 35–37]. Regarding the international collaboration, this emphasizes the significance of global networking and its impact on research output [38–41].

**Table 1:** Top 10 most productive countries

SCR	Country	Number of documents (%)	*h*-index	Average citations per document	No. of collaborative countries	No. of publications from collaboration
1st	USA	548 (57.14)	48	20.4	26	100
2nd	UK	69 (6.67)	15	11.5	22	35
3rd	Canada	46 (4.80)	17	35.3	5	14
3rd	Germany	46 (4.80)	13	15	15	16
5th	Australia	44 (4.59)	13	18.9	4	12
6th	Taiwan	43 (4.48)	11	13.9	6	12
7th	South Korea	36 (3.75)	13	16.2	1	16
8th	China	32 (3.34)	11	14.7	7	17
9th	Turkey	26 (2.71)	7	3.4	4	3
10th	Netherlands	25 (2.61)	8	17	6	11

*SCR* Standard competition ranking

Table 2 lists the top 10 most productive journals with their IF. The major publication outlets for research related to social media in psychology include *Computers in Human Behavior* (n = 375), *Cyberpsychology Behavior and Social Networking* (n = 134), the *Journal of Adolescent Health* (n = 30), *Personality and Individual Differences* (n = 25), and *American Behavioral Scientist* (n = 23).

**Table 2:** Ten most active journals according to the number of publications related to social media in psychology

SCR	Journal/periodical	Number of publications (%)	IF^a^
1st	*Computers in Human Behavior*	375 (39.10)	2.880
2nd	*Cyberpsychology Behavior and Social Networking*	134 (13.97)	2.188
3rd	*Journal of Adolescent Health*	30 (3.13)	3.838
4th	*Personality and Individual Differences*	25 (2.61)	1.964
5th	*American Behavioral Scientist*	23 (2.40)	1.907
6th	*Annals of Behavioral Medicine*	16 (1.67)	4.195
7th	*Psychological Reports*	12 (1.25)	0.414
7th	*Social Behavior and Personality*	12 (1.25)	0.366
9th	*Psychological Science*	9 (0.94)	5.476
10th	*Social Psychological and Personality Science*	8 (0.83)	2.325

*SCR* Standard competition ranking, *IF* Impact factor

^a^The impact factor was reported according to journal citation reports (JCR) 2015

**Fig. 3:**
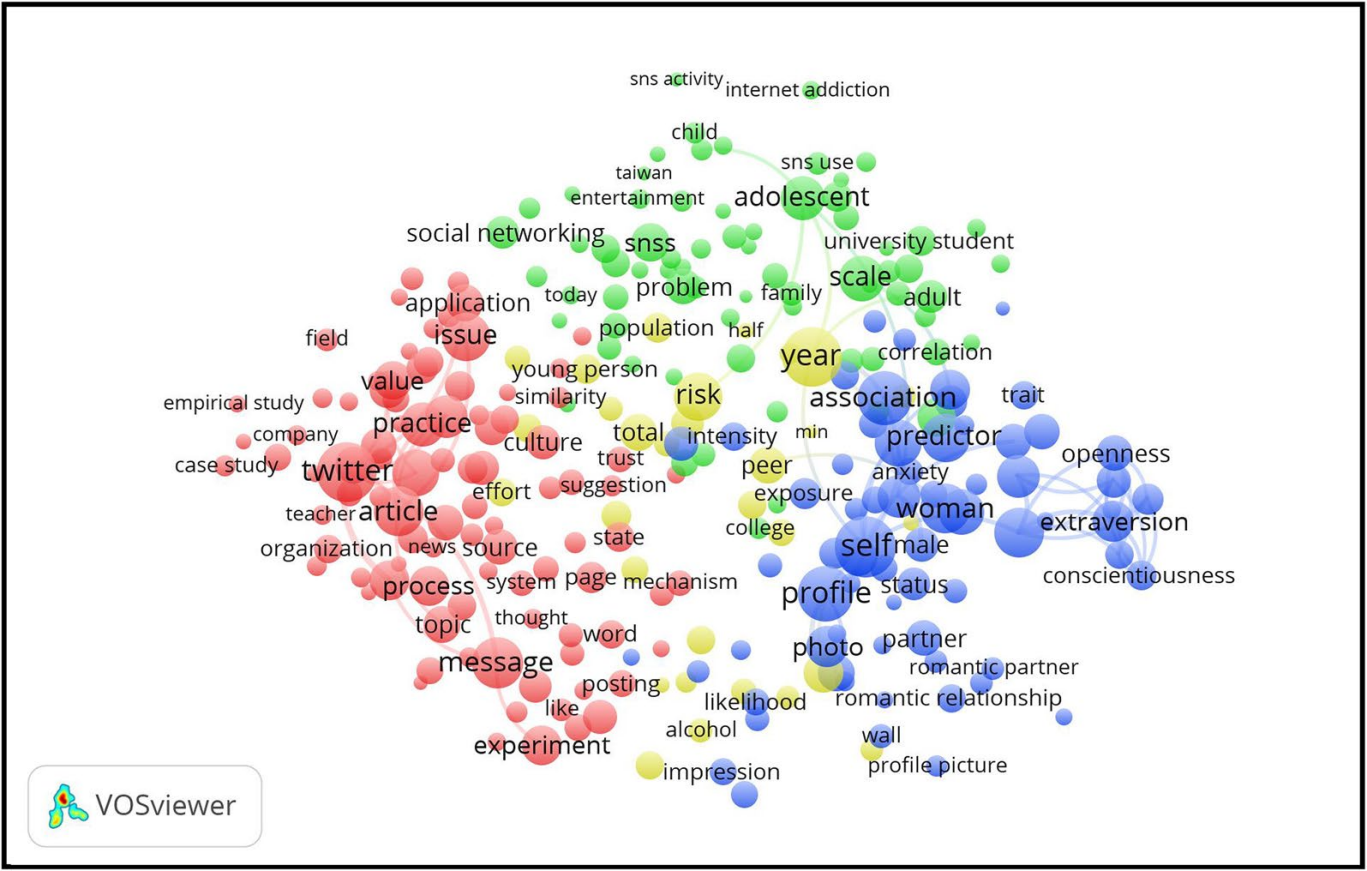
High-frequency terms in the titles and abstracts in publications related to social media in psychology with research topics indicated

The scientific landscape of main research areas related to social media in psychology is presented in Fig. 3, based on the retrieved publications terms co-occurrence network from the retrieved publications. The most important research areas related to social media in psychology were personality psychology, experimental psychology, psychological risk factors, and developmental psychology. Based on the map, the four main clusters (denoted by the green, blue, red, and yellow colours) were characterised by the most commonly used terms in the research related to social media in the psychology field. Green coloured cluster represented terms related to the developmental psychology topic, such as “child”, “adolescent”, or “adult”; blue coloured cluster represented terms related to personality psychology, such as “extraversion”, “openness”, or “romantic”; red coloured cluster represented terms related to experimental psychology, such as “empirical”, “experiment”, or “mechanism”; and yellow coloured cluster represented terms related to psychological risk factors, such as “risk” or “alcohol”.

Table 3 lists the 10 most cited among these papers. The 10 most cited articles were published in 4 journals [42–51]. The most-cited article, by Pempek et al., which investigated how much, why, and how college students use social networking sites, was published in the *Journal of Applied Developmental Psychology* in 2009 and has been cited 426 times [46]. The most recent manuscript in the top 10, by Lin and Lu, which investigated the factors affecting user’s joining social networking sites, was published in 2011 and has been cited 241 times [44].

**Table 3:** Ten top-cited publications for research related to social media in psychology

SCR	Title	Authors	Source title	Publication year	Total citations	Average per year
1st	College students’ social networking experiences on Facebook	Pempek et al. [46]	*Journal of Applied Developmental Psychology*	2009	426	47.33
2nd	Social capital, self-esteem, and use of online social network sites: a longitudinal analysis	Steinfield et al. [49]	*Journal of Applied Developmental Psychology*	2008	408	40.8
3rd	Personality and motivations associated with Facebook use	Ross et al. [48]	*Computers in Human Behavior*	2009	386	42.89
4th	Identity construction on Facebook: digital empowerment in anchored relationships	Zhao et al. [51]	*Computers in Human Behavior*	2008	327	32.7
5th	MySpace and Facebook: applying the uses and gratifications theory to exploring friend-networking sites	Raacke and Bonds-Raacke [47]	*Cyberpsychology & Behavior*	2008	313	31.3
6th	Who interacts on the Web?: The intersection of users’ personality and social media use	Correa et al. [43]	*Computers in Human Behavior*	2010	282	35.25
7th	Online and offline social networks: use of social networking sites by emerging adults	Subrahmanyam et al. [50]	*Journal of Applied Developmental Psychology*	2008	275	27.5
8th	Being immersed in social networking environment: Facebook groups, Uses and Gratifications, and Social Outcomes	Park et al. [45]	*Cyberpsychology & Behavior*	2009	263	29.22
9th	Why people use social networking sites: an empirical study integrating network externalities and motivation theory	Lin and Lu [44]	*Computers in Human Behavior*	2011	241	34.43
9th	Facebook profiles reflect actual personality, not self-idealization	Back et al. [42]	*Psychological Science*	2010	241	30.12

*SCR* Standard competition ranking

**Fig. 4:**
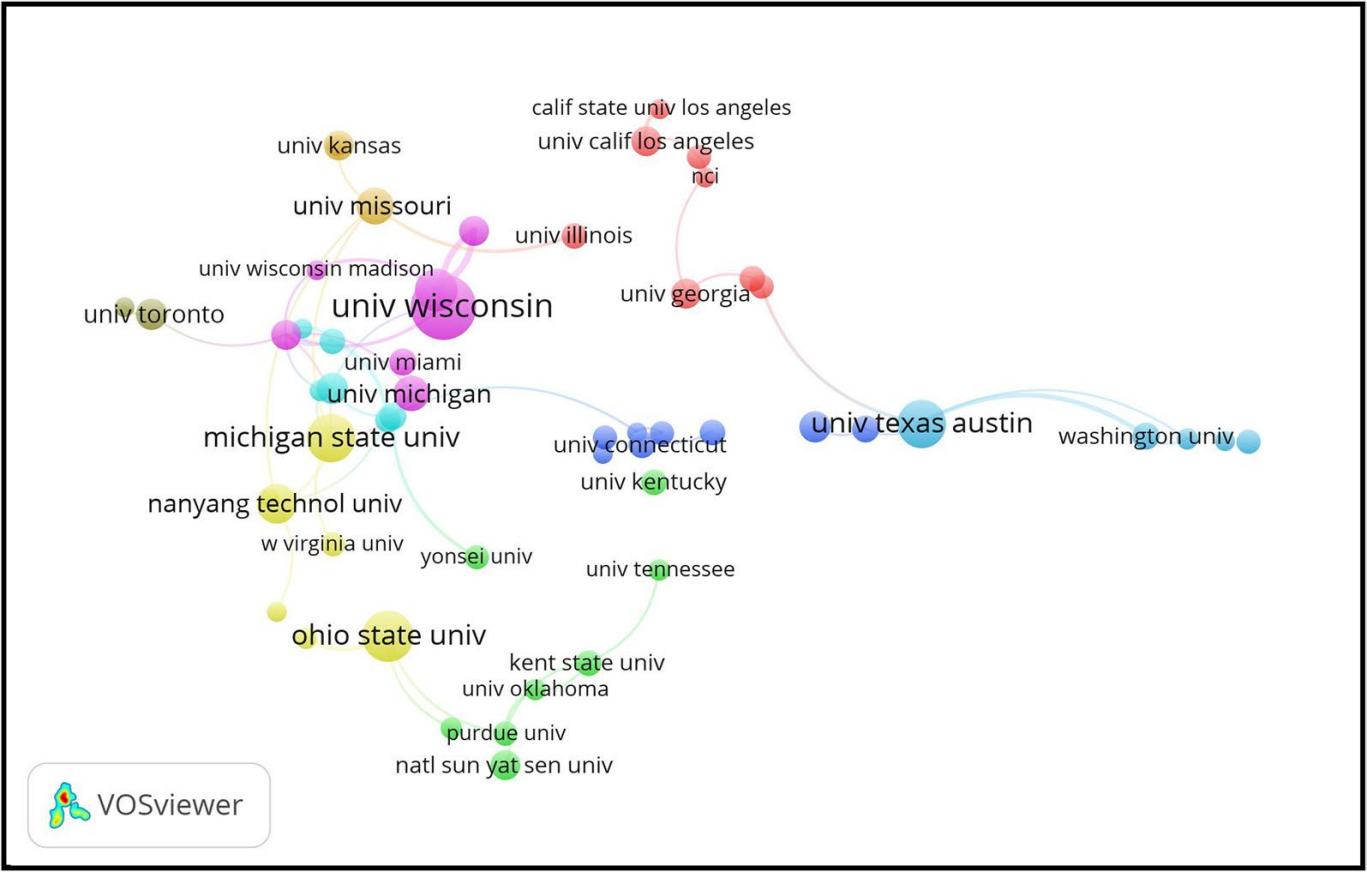
Combined mapping and clustering of productive institutes

Table 4 shows the top 10 institutions based on the number of publications related to social media in the psychology field. It is worth noting that the *University of Wisconsin*–*Madison* ranked first in terms of the total publications (n = 39). *Ohio State University* (n = 25) was second to the *University of Wisconsin*–*Madison* in the total number of publications, followed by *Michigan State University* (n = 23) and the *University of Texas at Austin* (n = 23). Figure 4 shows the collaboration between the most prolific institutions.

**Table 4:** Top 10 most productive institutes according to the number of publications related to social media in psychology

SCR	Institute	Country	Number of publications (%)
1st	*University of Wisconsin*–*Madison*	USA	39 (4.07)
2nd	*Ohio State University*	USA	25 (2.61)
3rd	*Michigan State University*	USA	23 (2.40)
3rd	*University of Texas at Austin*	USA	23 (2.40)
5th	*University of Washington*	USA	19 (1.98)
6th	*Nanyang Technological University*	Singapore	17 (1.77)
7th	*University of Michigan*	USA	15 (1.56)
7th	*University of Missouri*	USA	15 (1.56)
8th	*Cornell University*	USA	11 (1.15)
8th	*University of Alabama*	USA	11 (1.15)
8th	*University of Toronto*	Canada	11 (1.15)

*SCR* Standard competition ranking

This bibliometric study represents the first concise analysis of the global publications related to social media in psychology and shows the benefits of bibliometric analysis for evaluating research productivity in a standardised way. There are some limitations, most of which have already been mentioned in previous bibliometric studies [52–56]. The first is that the study was centred only on the most used social network worldwide. The value of other social networking sites might also be evaluated in order to provide a more faithful representation of all the research activities in this field. In our study, we decided to include only commonly-known networking sites related to the field of social media; however, social media is a rapidly changing and growing field [57]. A second limitation is that our study was based on the WoS Core Collection database; thus, it is expected that publications published in non-WoS-cited journals may not have been included in the analysis. Thirdly, some publications regarding social media might have been published in non-psychology journals.

## Conclusions

Up to the authors’ best knowledge, this is the first ever bibliometric study to report worldwide activity in social media—related research in psychology field. Our study provides some novel insights useful for policy makers, researchers, and funders interested in advancing an evidence-based social media and psychology research agenda. International research collaborations and research networks should be encouraged to help prioritize social media—related psychology research particularly in countries with research capacities. Our findings provide baseline data for scholars and policy makers to recognize the bibliometric indicators in the current study as measures of research performance in social media for future policies and funding decisions. Finally, our study showed that bibliometric analysis is a good methodological tool to map published literature in a particular subject and to pin point research gaps in that subject.

## Abbreviations

WoS: web of science
IF: impact factor

## References

[1] Kim W, Jeong O-R, Lee S-W (2010). On social Web sites. Inf syst.

[2] Perrin A. Social media usage: 2005–2015. Pew Research Center 2015; http://www.pewinternet.org/2015/10/08/social-networking-usage-2005-2015/. Accessed date 12 Nov 2017.

[3] Ellison NB (2007). Social network sites: definition, history, and scholarship. J Comput Mediat Commun.

[4] Dery K, Tansley C, Hafermalz E (2014). Hiring in the age of social media: new rules, new game. Univ Auckland Bus Rev.

[5] Knight-McCord J, Cleary D, Grant N, Herron A, Lacey T, Livingston T, Emanuel R (2016). What social media sites do college students use most?. J Undergrad Ethnic Minor Psycho.

[6] Best P, Manktelow R, Taylor B (2014). Online communication, social media and adolescent wellbeing: a systematic narrative review. Child Youth Serv Rev.

[7] Won HH, Myung W, Song GY, Lee WH, Kim JW, Carroll BJ, Kim DK (2013). Predicting national suicide numbers with social media data. PLoS ONE.

[8] Valenzuela S, Halpern D, Katz JE (2014). Social network sites, marriage well-being and divorce: survey and state-level evidence from the United States. Comput Human Behav.

[9] Clayton RB, Nagurney A, Smith JR (2013). Cheating, breakup, and divorce: is Facebook use to blame?. Cyberpsychol Behav Soc Netw.

[10] Clayton RB (2014). The third wheel: the impact of Twitter use on relationship infidelity and divorce. Cyberpsychol Behav Soc Netw.

[11] Lukacs V, Quan-Haase A (2015). Romantic breakups on Facebook: new scales for studying post-breakup behaviors, digital distress, and surveillance. Inf Commun Soc.

[12] Basak E, Calisir F: Publication trends in Facebook: A scientometric study. In *International Conference on Trends in Economics, Humanities and Management (ICTEHM’15) March 27*-*28, 2015 2015; Singapore*; 2015:170-173.

[13] Gupta R, Kumar N, Gupta B (2015). A bibliometric assessment of global literature on “Facebook and Libraries” during 2006–14. Inf Stud.

[14] Haustein S, Costas R, Lariviere V (2015). Characterizing social media metrics of scholarly papers: the effect of document properties and collaboration patterns. PLoS ONE.

[15] Gan C, Wang W (2015). Research characteristics and status on social media in China: a bibliometric and co-word analysis. Scientometrics.

[16] Ho YS, Hartley J (2016). Classic articles in psychology in the science citation index expanded: a bibliometric analysis. Br J Psychol.

[17] Piotrowski C (2016). Mapping the research domain in the field of applied psychology: a bibliometric analysis of the emerging literature. J Indian Acad Appl Psychol.

[18] Allik J (2013). Personality psychology in the first decade of the new millennium: a bibliometric portrait. Eur J Pers.

[19] Cañas-Guerrero I, Mazarrón FR, Pou-Merina A, Calleja-Perucho C, Díaz-Rubio G (2013). Bibliometric analysis of research activity in the “Agronomy” category from the Web of Science, 1997–2011. Eur J Agron.

[20] Hew J-J (2017). Hall of fame for mobile commerce and its applications: a bibliometric evaluation of a decade and a half (2000–2015). Telemat Inform.

[21] Falagas ME, Pitsouni EI, Malietzis GA, Pappas G (2008). Comparison of PubMed, scopus, Web of science, and google scholar: strengths and weaknesses. FASEB J.

[22] Kulkarni AV, Aziz B, Shams I, Busse JW (2009). Comparisons of citations in Web of science, scopus, and google scholar for articles published in general medical journals. JAMA.

[23] Thomson Reuters. Web of Science Core Collection. 2017; http://thomsonreuters.com/en/products-services/scholarly-scientific-research/scholarly-search-and-discovery/web-of-science-core-collection.html. Accessed 7 Jan 2017.

[24] Thomson Reuters. 2015 Journal Citation Reports^®^ 2016 ; https://jcr.incites.thomsonreuters.com/. Accessed 4 Jan 2017.

[25] Meng J, Martinez L, Holmstrom A, Chung M, Cox J (2017). Research on social networking sites and social support from 2004 to 2015: a narrative review and directions for future research. Cyberpsychol Behav Soc Netw.

[26] Shi J, Poorisat T, Salmon CT (2018). The use of social networking sites (SNSs) in health communication campaigns: review and recommendations. Health Commun.

[27] Hamm MP, Chisholm A, Shulhan J, Milne A, Scott SD, Klassen TP, Hartling L (2013). Social media use by health care professionals and trainees: a scoping review. Acad Med.

[28] Li J (2013). Privacy policies for health social networking sites. J Am Med Inform Assoc.

[29] Moorhead SA, Hazlett DE, Harrison L, Carroll JK, Irwin A, Hoving C (2013). A new dimension of health care: systematic review of the uses, benefits, and limitations of social media for health communication. J Med Internet Res.

[30] Ventola CL (2014). Social media and health care professionals: benefits, risks, and best practices. Phram Ther.

[31] Antheunis ML, Tates K, Nieboer TE (2013). Patients’ and health professionals’ use of social media in health care: motives, barriers and expectations. Patient Educ Couns.

[32] Bielsa S, Porcel JM (2016). Trends in pleural effusion research. Pleura.

[33] Yun EJ, Yoon DY, Kim BN, Min KJ, Kim BY, Ku YJ (2015). Endovascular treatment for extracranial carotid stenosis: a 10-year bibliometric analysis. Vasc Endovascular Surg.

[34] Vioque J, Ramos JM, Navarrete-Munoz EM, Garcia-de-la-Hera M (2010). A bibliometric study of scientific literature on obesity research in PubMed (1988–2007). Obes Rev.

[35] Zyoud SH, Al-Jabi SW, Sweileh WM, Awang R, Waring WS (2015). Bibliometric profile of the global scientific research on methanol poisoning (1902–2012). J Occup Med Toxicol.

[36] Bruggmann D, Pulch K, Klingelhofer D, Pearce CL, Groneberg DA (2017). Ovarian cancer: density equalizing mapping of the global research architecture. Int J Health Geogr.

[37] Gotting M, Schwarzer M, Gerber A, Klingelhofer D, Groneberg DA (2017). Pulmonary hypertension: scientometric analysis and density-equalizing mapping. PLoS ONE.

[38] Gal D, Glanzel W, Sipido KR (2017). Mapping cross-border collaboration and communication in cardiovascular research from 1992 to 2012. Eur Heart J.

[39] Orwat MI, Kempny A, Bauer U, Gatzoulis MA, Baumgartner H, Diller GP (2015). The importance of national and international collaboration in adult congenital heart disease: a network analysis of research output. Int J Cardiol.

[40] Khan A, Choudhury N, Uddin S, Hossain L, Baur LA (2016). Longitudinal trends in global obesity research and collaboration: a review using bibliometric metadata. Obes Rev.

[41] Adams J (2012). Collaborations: the rise of research networks. Nature.

[42] Back MD, Stopfer JM, Vazire S, Gaddis S, Schmukle SC, Egloff B, Gosling SD (2010). Facebook profiles reflect actual personality, not self-idealization. Psychol Sci.

[43] Correa T, Hinsley AW, de Zúñiga HG (2010). Who interacts on the Web?: the intersection of users’ personality and social media use. Comput Human Behav.

[44] Lin K-Y, Lu H-P (2011). Why people use social networking sites: an empirical study integrating network externalities and motivation theory. Comput Human Behav.

[45] Park N, Kee KF, Valenzuela S (2009). Being immersed in social networking environment: Facebook groups, uses and gratifications, and social outcomes. Cyberpsychol Behav.

[46] Pempek TA, Yermolayeva YA, Calvert SL (2009). College students’ social networking experiences on Facebook. J Appl Dev Psychol.

[47] Raacke J, Bonds-Raacke J (2008). MySpace and Facebook: applying the uses and gratifications theory to exploring friend-networking sites. Cyberpsychol Behav.

[48] Ross C, Orr ES, Sisic M, Arseneault JM, Simmering MG, Orr RR (2009). Personality and motivations associated with Facebook use. Comput Human Behav.

[49] Steinfield C, Ellison NB, Lampe C (2008). Social capital, self-esteem, and use of online social network sites: a longitudinal analysis. J Appl Dev Psychol.

[50] Subrahmanyam K, Reich SM, Waechter N, Espinoza G (2008). Online and offline social networks: use of social networking sites by emerging adults. J Appl Dev Psychol.

[51] Zhao S, Grasmuck S, Martin J (2008). Identity construction on Facebook: digital empowerment in anchored relationships. Comput Human Behav.

[52] Zyoud SH, Waring WS, Al-Jabi SW, Sweileh WM, Rahhal B, Awang R (2016). Intravenous lipid emulsion as an antidote for the treatment of acute poisoning: a Bibliometric Analysis of Human and Animal Studies. Basic Clin Pharmacol Toxicol.

[53] Zyoud SH (2016). Global research trends of Middle East respiratory syndrome coronavirus: a bibliometric analysis. BMC Infect Dis.

[54] Zyoud SH, Waring WS, Al-Jabi SW, Sweileh WM (2017). Global research production in glyphosate intoxication from 1978 to 2015: a bibliometric analysis. Hum Exp Toxicol.

[55] Sweileh WM, AbuTaha AS, Sawalha AF, Al-Khalil S, Al-Jabi SW, Zyoud SH (2016). Bibliometric analysis of worldwide publications on multi-, extensively, and totally drug—resistant tuberculosis (2006–2015). Multidiscip Respir Med.

[56] Zyoud SH (2016). Dengue research: a bibliometric analysis of worldwide and Arab publications during 1872–2015. Virol J.

[57] Appio FP, Martini A, Massa S, Testa S (2016). Unveiling the intellectual origins of social media-based innovation: insights from a bibliometric approach. Scientometrics.

